# Bluetongue in Belgium, 2006

**DOI:** 10.3201/eid1304.061136

**Published:** 2007-04

**Authors:** Jean-François Toussaint, Corinne Sailleau, Jan Mast, Philippe Houdart, Guy Czaplicki, Lien Demeestere, Frank VandenBussche, Wesley van Dessel, Nesya Goris, Emmanuel Bréard, Lotfi Bounaadja, Etienne Thiry, Stephan Zientara, Kris de Clercq

**Affiliations:** * Veterinary and Agrochemical Research Centre, Brussels, Belgium; † Agence Française de Sécurité Sanitaire des Aliments, Maisons-Alfort, France; ‡ Federal Agency for the Safety of the Food Chain, Brussels, Belgium; § Association Régionale de Santé et d’Identification Animales, Loncin, Belgium; ¶ University of Liège, Liège, Belgium

**Keywords:** Bluetongue, Bluetongue virus, Europe, Belgium, arbovirus, cattle, sheep, real-time PCR, dispatch

## Abstract

Bluetongue has emerged recently in Belgium. A bluetongue virus strain was isolated and characterized as serotype 8. Two new real-time reverse transcription–quantitative PCRs (RT-qPCRs) that amplified 2 different segments of bluetongue virus detected this exotic strain. These 2 RT-qPCRs detected infection earlier than a competitive ELISA for antibody detection.

Bluetongue is a noncontagious disease caused by an orbivirus of the family *Reoviridae*. The bluetongue virus (BTV) serogroup consists of 24 serotypes. BTV is transmitted by arthropods of the genus *Culicoides* and its distribution worldwide is restricted to regions that contain competent vectors (*1*). An outbreak of bluetongue was reported and confirmed in the Netherlands on August 17, 2006 (*2*). Belgium reported its first cases of bluetongue 1 day later, and Germany and France reported outbreaks on August 21, 2006, and August 31, 2006, respectively (*2*,*3*). We report detection and characterization of a BTV strain and an overview of laboratory test results 4 weeks after the onset of the outbreak.

## The Study

Twenty-one animals (16 cattle and 5 sheep) showing clinical signs suggestive of bluetongue were sampled by the Federal Agency for the Safety of the Food Chain on August 18, 2006, at 11 farms in northeastern Belgium. Two serologic tests that detect antibodies against the major serogroup antigen VP7 (bluetongue virus antibody competitive ELISA [cELISA]; Veterinary Medical Research and Development Inc., Pullman, WA, USA and competitive vp7 bluetongue kit; IDVET, Montpellier, France) identified 21 virus-positive animals. Two newly developed and validated reverse transcription–quantitative PCRs (RT-qPCRs) that detected BTV strains representing the 24 serotypes (*4*) were then conducted to determine whether these seropositive animals also had viral RNA. The first assay (RT-qPCR_S1), which amplified a 357-nt fragment in segment 1, detected virus in erythrocytes of the 21 seropositive animals (mean cycle threshold [Ct] value 29.0). The second assay (RT-qPCR_S5), which amplified a 94-nt fragment in segment 5, detected virus in the same 21 seropositive animals (mean Ct value 26.5). The 2 serologic tests and the 2 molecular assays detected BTV in 21 animals from 11 Belgian farms within 14 hours.

**Figure 1:**
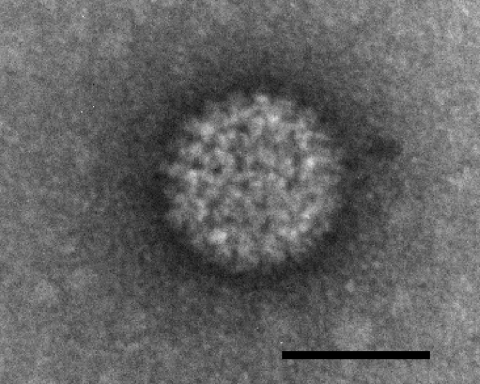
Negatively stained bluetongue viruslike particle that caused a cytopathic effect in BHK-21 cells. Scale bar = 50 nm.

Virus isolation was conducted on August 18, 2006, by injection of blood from infected sheep into 11-day-old embryonated chicken eggs, followed by passage on BHK-21 cells (ATCC-CCL10) as previously described (*5*). The specificity of the cytopathic effect observed 48 hours after passage on BHK-21 cells was confirmed by RT-qPCR and electron microscopy (Figure 1) after fixation and negative staining as previously described (*6*). Two virus neutralization tests were conducted on 2 virus strains isolated by the Belgian and the French reference laboratories at 2 Belgian farms 30 km apart. The 2 BTV isolates were completely neutralized with reference serum for serotype 8. Each strain was also partially neutralized by reference serum against serotype 18, which confirmed cross-neutralization between serotypes 8 and 18 (*7*).

From August 19, 2006, to September 14, 2006, the study farms were screened for animals with clinical signs of bluetongue. Blood samples were tested by serologic tests or RT-qPCR. For cattle, 97 (68%) of 142 samples had antibodies to BTV and 32 (78%) of 41 samples contained viral RNA (Table 1). However, for sheep, only 23 (29%) of 79 samples had antibodies to BTV and 15 (45%) of 33 samples contained viral RNA. Other diseases that cause similar signs might explain this lower frequency in sheep. Contagious ecthyma was diagnosed by using PCR and electron microscopy for several sheep that showed bluetonguelike signs but did not have antibodies to BTV or viral RNA.

**Table 1:** Bluetongue virus infection in cattle and sheep with bluetonguelike clinical signs by IDVET cELISA and RT-qPCR, Belgium, 2006*

Test	Cattle	Sheep
cELISA		
No. negative	45	56
No. positive	97	23
% Confirmed cases	68	29
RT-qPCR		
No. negative	9	18
No. positive	32	15
% Confirmed cases	78	45

*cELISA, competitive ELISA;RT-qPCR, reverse transcription–quantitative PCR.

Agreement between cELISA and RT-qPCR results was analyzed for 124 animals (Table 2). One sample negative by RT-qPCR_S1 and RT-qPCR_S5 was positive by cELISA. Although this result might reflect lack of specificity of the cELISA, elimination of BTV RNA from the animal several weeks after being infected cannot be ruled out. A false-negative result in the RT-qPCR is unlikely because 1) 2 different RT-qPCRs that amplified 2 different segments were used, 2) the quality of the RNA was confirmed by a third RT-qPCR that quantified mRNA of β-actin, and 3) both RT-qPCRs are highly sensitive (they can detect 0.01 infectious doses of virus) (*4*). False-positive results were not observed with the IDVET cELISA when we analyzed 650 negative serum samples from artificial insemination centers and field samples collected from Belgian livestock in 2004 and 2005. Thus, the specificity of the cELISA is >99.8%. Seven animals with bluetonguelike clinical signs were positive according to each RT-qPCR but negative according to the cELISA (Table 2). These results support the finding that RT-qPCR can be used to detect viral RNA in infected animals before antibodies are detectable. The clinical signs indicative of a recent infection support this finding.

**Table 2:** Agreement between results of IDVET cELISA and RT-qPCR_S5 for bluetongue virus infection, Belgium, 2006*

cELISA result	RT-qPCR result†	
Negative	Positive
Negative	75	7
Positive	1	41

*cELISA, competititve ELISA; RT-qPCR, reverse transcription–quantitative PCR. †Samples with different results in cELISA and RT-qPCR_S5 were retested with RT qPCR_S1 (*4*). This latter test always confirmed the result of the RT-qPCR_S5.

**Figure 2:**
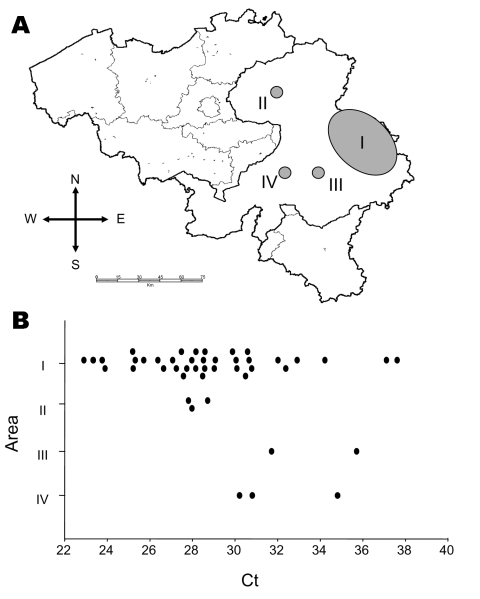
A) Distribution of outbreaks of bluetongue (shaded areas) reported in Belgium from August 18 through September 14, 2006. Area I is where the disease was initially detected. B) Cycle threshold (Ct) values observed in different zones as a result of conducting reverse transcription–quantitative PCR_S5 on individual blood samples.

On September 14, 2006 (4 weeks after the first identification of BTV in Belgium) as many as 84 Belgian farms had at least 1 BTV-infected animal. The maximal distance between herds in this study was ≈110 km (Figure 2A). Most outbreaks were confirmed in the area where the disease was initially detected (area I, Figure 2). Most (64%) infected animals showed a high virus load with individual Ct values <30. Of the remaining animals, 30% had moderate virus loads (Ct values 30–35) and 6% had Ct values >35. The high Ct values for the latter animals might have remained undetected had pooled blood samples been analyzed. Thus, results of pooled samples need to be validated before being used for diagnosis. Distribution of Ct values differed slightly, depending on the origin of the animals. None of the animals from zones III and IV showed a Ct value <30, whereas all animals from zone II showed low Ct values, which are indicative of high virus loads. Lower virus loads and acute clinical signs in animals from zones III and IV might indicate onset of infection. However, we cannot rule out decreased infection in these animals because they also had positive serologic results that indicated infection for at least 4–5 days. Further epidemiologic studies are required before conclusions can be drawn on the evolution of these epidemics.

## Conclusions

Bluetongue has emerged in some countries of northern Europe. BTV has been detected and its isolation and characterization have considerably progressed in the first weeks of the epidemics. Results of virus neutralization tests for 2 Belgian isolates and molecular characterization of the Dutch BTV strain by the community reference laboratory (*8*) indicate that BTV serotype 8 is present in Belgium and the Netherlands. Although this observation suggests 1 serotype circulating in northern Europe after a common virus introduction, it must be confirmed by detailed epidemiologic studies. The mechanism of introduction of BTV strain serotype 8 is unknown. Northward spread of bluetongue in Europe has been correlated with climate warming (*9*). However, BTV serotype 8 has not been found in the Mediterranean basin.

One characteristic of the current epidemics of bluetongue is the severity of clinical signs reported in cattle (*10*). The present results also demonstrate that clinical signs observed in cattle are more specific than those observed in sheep. Confusing clinical signs in sheep underline the need for developing diagnostic tests to discriminate between bluetongue and other confounding diseases such as contagious ecthyma, border disease, and foot-and-mouth disease. Our results also indicate the usefulness of RT-qPCR, which detected viral RNA in recently infected animals with clinical signs of bluetongue but no detectable antibodies to BTV. The RT-qPCR and ELISA are independent but complementary tests because they detect viral RNA and virus-specific antibodies, respectively. These tests indicated that an outbreak of bluetongue was occurring in Belgium. Despite high sensitivity of RT-qPCR (*4*), our results suggest that using this test with pooled samples might not detect animals with low viral loads. This possibility should be explored and validated by testing individual and pooled samples. RT-PCR–positive results in animals that are no longer infectious (*11*) should also be considered before deciding whether pooled samples are acceptable.
